# Mechanisms of Action and Limitations of Monoclonal Antibodies and Single Chain Fragment Variable (scFv) in the Treatment of Cancer

**DOI:** 10.3390/biomedicines11061610

**Published:** 2023-06-01

**Authors:** Cynthia Rodríguez-Nava, Carlos Ortuño-Pineda, Berenice Illades-Aguiar, Eugenia Flores-Alfaro, Marco Antonio Leyva-Vázquez, Isela Parra-Rojas, Oscar del Moral-Hernández, Amalia Vences-Velázquez, Karen Cortés-Sarabia, Luz del Carmen Alarcón-Romero

**Affiliations:** 1 Laboratorio de Investigación en Citopatología e Histoquímica, Universidad Autónoma de Guerrero, Chilpancingo de los Bravo 39070, Mexico; 2 Laboratorio de Investigación en Inmunobiología y Diagnóstico Molecular, Universidad Autónoma de Guerrero, Chilpancingo de los Bravo 39070, Mexico; 3 Laboratorio de Proteínas y Ácidos Nucleicos, Universidad Autónoma de Guerrero, Chilpancingo de los Bravo 39070, Mexico; 4 Laboratorio de Investigación en Biomedicina Molecular, Universidad Autónoma de Guerrero, Chilpancingo de los Bravo 39070, Mexico; 5 Laboratorio de Investigación en Epidemiología Clínica y Molecular, Universidad Autónoma de Guerrero, Chilpancingo de los Bravo 39070, Mexico; 6 Laboratorio de Investigación en Obesidad y Diabetes, Universidad Autónoma de Guerrero, Chilpancingo de los Bravo 39070, Mexico; 7 Laboratorio de Virología, Universidad Autónoma de Guerrero, Chilpancingo de los Bravo 39070, Mexico

**Keywords:** cancer, monoclonal antibodies, scFv, treatment, mechanism of action

## Abstract

Monoclonal antibodies are among the most effective tools for detecting tumor-associated antigens. The U.S. Food and Drug Administration (FDA) has approved more than 36 therapeutic antibodies for developing novel alternative therapies that have significant success rates in fighting cancer. However, some functional limitations have been described, such as their access to solid tumors and low interaction with the immune system. Single-chain variable fragments (scFv) are versatile and easy to produce, and being an attractive tool for use in immunotherapy models. The small size of scFv can be advantageous for treatment due to its short half-life and other characteristics related to the structural and functional aspects of the antibodies. Therefore, the main objective of this review was to describe the current situation regarding the mechanisms of action, applications, and limitations of monoclonal antibodies and scFv in the treatment of cancer.

## 1. Introduction

Cancer represents one of the leading causes of death worldwide, despite advances in diagnosis and treatment. Several side effects, such as relapse and resistance to therapy, have been associated with the non-specificity of conventional therapies (such as chemotherapy and radiotherapy) [1]. Therefore, the cancer biomedical research community has been focused on searching for specific novel molecules related to each type of cancer for a more personalized therapeutic approach [2]. Antibodies are molecules capable of recognizing tumor cells due to their specific recognition of tumoral antigens. In addition, they can be used to target drugs for immune system activation and early tumor detection [3]. Despite all the promising applications accomplished using monoclonal antibodies (mAb), they also have some therapeutic disadvantages, such as their difficulty in penetrating to solid tumors due to the complexity of the tumor microenvironment. To overcome these difficulties, advances in genetic engineering have enabled the creation of different antibody formats by modifying or eliminating the Fc region. One of the most used is scFv, a novel short format antibody capable of recognizing the target antigen but lacking the fragmented crystallizable (Fc) region. ScFv represents a basic functional unit for developing antibodies and more complex molecules, such as bi-, tri-, tetra-specific, and immunotoxins. However, the small size of these molecules reduces their half-life in blood; therefore, more complex structures are needed to achieve therapeutic effects [4].

## 2. Cancer Overview

According to the World Health Organization (WHO), in 2019, cancer was the second cause of death in patients younger than 70 years old from 112 countries. In 2020, 19.3 million new cases (18.1 million cases of non-melanoma skin cancer were excluded) and almost 10 million deaths by cancer were reported. About 70% of those deaths were in low- and middle-income countries. In 2040, the number of cases is expected to increase to 28.4 million, representing an increase of 47%. GLOBOCAN reported that the ten most diagnosed types of cancer were: breast (in females) (11.7%), lung (11.4%), colorectal (19%), prostate (7.3%), and stomach (5.6%). However, in terms of mortality, lung cancer (18%), colorectal (9.4%), liver (8.3%), stomach (7.7%), and breast cancer (6.9%) were the most common. If the number of cases was classified according to sex, in men, lung, prostate, and colorectal cancers represent the most frequent, whereas liver and colorectal had the highest mortality rates. In females, breast cancer ranks first in causes of death by cancer, followed by cervical cancer [5].

Cancer is defined as the alteration in the cellular growth of normal cells and can originate in any organ. Tumor cells are characterized by the loss of control of cellular division. According to the WHO, metastasis is typically the actual cause of death due to the multiplication and invasion of adjacent organs by neoplastic cells [6]. Cancer is commonly detected when the number of cells reaches one million or when the tumor size has reached one centimeter, except for in the blood and bone marrow (leukemia and lymphomas), as these do not form solid structures [7]. This process results in the loss of function in normal cells and the gain of malignant characteristics (tumorigenesis) that includes dedifferentiation, increased proliferation, metastasis, apoptosis, and immunosurveillance inhibition, and changes in the metabolism and epigenetic functions (i.e., hallmarks of cancer) [8].

The risk factors for cancer development have been grouped into the following categories: tobacco use; infectious agents; alcohol consumption; ultraviolet or ionizing radiation; obesity; dietary carcinogens; air and water pollution; drugs (diethylstilbestrol and phenacetin); and exposure to occupational carcinogens, along with other risk factors, such as genetics, poor diet, lack of physical activity, poor immune status, and age, which perform an essential role in the development of cancer [9]. However, the WHO stated that many types of cancer have a high chance of being cured if they are diagnosed in the early stages. Due to the worldwide relevance of cancer, the search for alternatives that could improve the diagnosis, treatment, and research has been promoted. Several tools that could identify the target molecules in cancer have been produced, including antibodies that target essential proteins during oncogenic development [10].

## 3. Monoclonal Antibodies: Structure and Function

Immunoglobulins (Ig) or antibodies have a molecular weight of around 150 kDa and are produced by plasma or B-cells. Structurally, they contain two functional parts: Fc or a crystallizable region (associated with the effector mechanism) and a fragmented antigen-binding (Fab) region for the recognition of the target antigen. The two functional regions of the antibody are composed of two polypeptide chains: two light and two heavy chains, joined by disulfide bonds that confer stability and rigidity. The heavy chains have one variable domain (VH) and three constant domains (CH1, CH2, and CH3). The light chain has one variable domain (VL) and one constant domain (CL). The Fab region is made up of VH and CH1 together with VL and CL, while the Fc region consists of two segments, CH2 and CH3. Antibodies also have post-translational modifications, such as glycosylation in the Fc domain, that stabilize and modulate the binding to Fc receptors [11].

### 3.1. Monoclonal Antibodies Production Methods

Monoclonal antibodies (mAbs) come from a single cellular clone that has been divided multiple times in order to produce antibodies against the same antigen. The most common antibody production method is based on the generation of hybridomas, which are cells derived from the fusion of spleen and myeloma cells. Hybridomas possess two fundamental abilities: produce antibodies and proliferate indefinitely [12]. The method consists of immunizing mice with the antigen, spleen extraction, and fusion with myeloma cells. Hybrids are cloned by limiting dilution to ensure the growth and proliferation of one single cell per well. Finally, the clone is expanded, and the antibody is purified from the culture medium and validated [13]. The generation of hybridomas has been the most common technique for mAb production, and this technique can be modified or changed for the specific production of therapeutic antibodies. The substitution of murine regions with human sequences and the preservation of the Fab region has resulted in chimeric antibodies (-ximab), while the substitution of the Fc and Fab domains with human sequences and the preservation of the murine hypervariable regions (CDR) has resulted in antibodies that are close to the human version. Finally, the production of fully human antibodies (-zumab) has been achieved by using transgenic mice and molecular biology techniques [14].

For the generation of chimeric, humanized, and fully human antibodies, different genetic engineering techniques have been developed. These techniques have been based on the use of transgenic animals and the amplification of genes that encode for the antibody from B-cells or hybridomas. The process began starts with RNA extraction, cDNA synthesis by reverse transcription, then the subsequent amplification of the heavy and light chain encoding genes by PCR. Afterward, genes were cloned into different expression systems, such as bacteria, yeasts, and mammal cells. At this point, several formats could be obtained using specific regions derived from the antibody to facilitate large-scale production for commercial and therapeutic purposes [15,16,17].

### 3.2. Monoclonal Antibodies in Cancer Treatment

The use of monoclonal antibodies has been considered a novel treatment against cancer in conjunction with conventional therapies, such as surgery, radiation, and chemotherapy. The main advantages of mAbs are their mechanism of action, which could promote the death of tumor cells by recognizing the tumor-associated antigens (TAA) and the stimulation of long-lasting antitumoral activities without any effect on healthy cells [14]. TAA are proteins overexpressed on the surface of tumor cells, including mutated proteins and those with post-translational modifications [18,19].

Since the approval of the first commercial monoclonal antibody (Rituximab) by the U.S. FDA in 1997, many antibodies have been developed and approved (Table 1) [20]. Rituximab is a chimeric antibody that targets the loops H1, H2, H3, and L3 (169-PANPSE-174 and 183-CYSIQ-187 regions) of the extracellular domain of CD20 [21,22]. CD20 is expressed in B-cell during maturation and B-cell neoplastic cells, and it is lost after differentiation to plasmatic cells. Due to the success of Rituximab in the treatment of non-Hodgkin’s lymphoma, other antibodies targeting CD20 were developed [23]. Additional research enabled the authorization of mAbs for more than one type of cancer; for example, Sacituzumab Govitecan was approved for the first time in 2020 for the treatment of solid tumors. Furthermore, it was recently approved for use in patients with metastatic or locally advanced urothelial cancer [24], triple-negative breast cancer [25], and HR-positive breast cancer [26]. In addition, it was proposed the combinations of various mAbs targeting different TAAs [27]. MAbs are biological reagents that can be modified, improved, and continuously evolved to enhance their efficacy in multiple types of cancer.

Therapeutic mAbs approved by the FDA target a special type of TAA, named differentiation clusters, which are overexpressed on the surface of lymphocytes (used in directed therapies against hematopoietic tumors), growth factors essential for the cellular proliferation in specific tissues (targets in the treatment of solid tumors) and transmembrane proteins involved in cellular adhesion (Nectin 4), signaling transduction (Trop2) and immunological checkpoints (PD-1/PD-L1) (Table 1). Based on the current successful antibodies and therapeutic targets, novel antibodies targeting different epitopes were developed. For example, Cetuximab, Necitumumab, and Panitumumab target the same TAA but different epitopes in domain III of EGFR, and they compete with EGF for the binding site in EGFR to block signaling and cellular proliferation. Panitumumab overlapped with the binding site of EGF in D355 and K443, whereas Cetuximab overlapped with the binding site in D355, Q408, H409, K433, and S468 [33]. However, due to the presence of structural mutations in the sequence of domain III in EGFR, a notable decrease in the recognition of these antibodies was observed [63]. The main reported mutations described in EGFR were the following: V441, S442, I462, S464L, G465R, I491M, K467T, K489, and S492R. These could be involved in the resistance to therapy due to their presence in epitopes recognized by Panitumumab and Cetuximab [31,64,65]. It was reported that Necitumumab could bind to EGFR, in addition to the mutation S492R, in the domain III of EGFR that conferred resistance to Cetuximab [66]. Additional studies were performed to analyze the presence of novel epitopes in the same domain to target novel antibodies and provide new alternatives in the case of resistance [42].

In addition to the homology of the target, all the aforementioned antibodies possess unique structural and functional characteristics. For example, among anti-EGFR, Cetuximab is an IgG1 mAb, and Panitumumab is an IgG2, and the structural difference was the size of the hinge (15 amino acids for IgG1 and 12 to IgG2) that was associated with flexibility [67]. It was reported that Cetuximab was capable of inducing the activation of cytotoxic T cells against tumor cells, while Panitumumab had a low binding affinity to CD16 and could not induce ADCC promoted by NK cells or cytotoxic T cells; however, it could induce cytotoxicity mediated by neutrophils and monocytes [33].

#### 3.2.1. Effector Mechanisms of mAb in Therapy

In mammals, antibodies have been classified into five classes: IgM, IgD, IgG, IgE, and IgA. The most commonly used isotype in cancer therapy is IgG [68]. The characteristic “Y” shape of antibodies has been associated with the basic unit of Ig. Antibodies can specifically recognize one defined antigen in Fab regions and perform its biological functions in the Fc region, which could then bind to cellular receptors in macrophages or mast cells or mediate cytotoxic activities by the complement or NK cells [69].

##### Blocking Signaling Pathways

MAbs can induce the death of tumor cells by blocking the signaling pathways associated with growth factor receptorsGrowth signaling and tumor survival could be interrupted when a mAb recognize by the Fab region to receptors for the growth factors and inactivates signaling pathways or blocks the of the ligand. For example, one of the most used targets with this mechanism was the receptor for the epidermal growth factor (EGFR) [70], which can be overexpressed in different types of cancer, such as colon, neck, and head, ovary, and lung, among others. It was reported that the activation of EGFR promoted an increase in the proliferation rate, migration, and cellular invasion, through the stimulation of the signaling pathways phosphoinositol 3-kinase (PI3K) and guanosine triphosphatase (GTPase) Ras [71].

**Figure 1:**
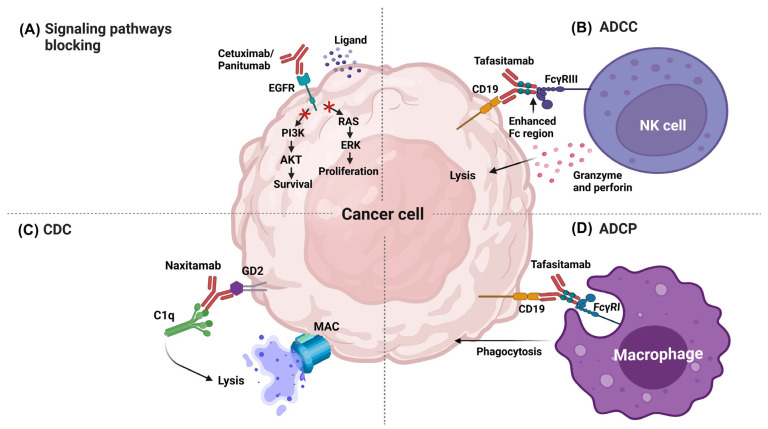
Effector mechanisms of therapeutic mAbs in cancer therapy. (**A**) Signaling pathway blocking. (**B**) Antibody-dependent cellular cytotoxicity. (**C**) Complement-dependent cytotoxicity. (**D**) Antibody-dependent cellular phagocytosis. AKT: Protein kinase B, ERK: extracellular signal-regulated kinase, C1q: complement component 1q, MAC: Membrane attack complex, FcγRIII: Fc-gamma receptor III, FcγRI: Fc-gamma receptor I. Image created in BioRender (www.biorender.com, accessed on 20 January 2023).

Some mAbs approved by the FDA act by blocking signaling pathways, such as Cetuximab and Panitumumab. Cetuximab was able to bind to EGFR and competitively inhibited the binding to the epidermal growth factor (EGF) and other ligands, which blocked the phosphorylation of EGFR induced by ligands and mitigated the activation of the signaling pathways related to cancer development [72] (Figure 1A). Panitumumab is an antagonist and induces the internalization of EGFR. The intracellular processes triggered by EGFR activation (e.g., dimerization, autophosphorylation, and signal transduction) were prevented using this mAb, which promoted an increase in the apoptotic rate and a reduction in the proliferation and angiogenesis of tumor cells [73].

##### Antibody-Dependent Cellular Cytotoxicity

Antibody-dependent cellular cytotoxicity (ADCC) is an effector function derived from the antibody binding to the tumor cell and immune cells. The variable regions of the antibody could bind to antigens in the tumor cell, and the Fc region could bind to the Fcγ receptors (FcγR) expressed in leukocytes; for example, FcγRIIIA expressed in natural killer (NK) cells promoted cellular destruction through the release of lytic factors [74]. Tafasitamab is one of the most recently approved therapeutic mAbs by the FDA; its target is CD19, a differentiation cluster successfully used as a target for other therapeutic antibodies, such as Loncastuximab and Blinatumomab. The expression of CD19 is limited to B-cells during maturation and is overexpressed in B-cell-associated tumors [75]. Tafasitamab contains modifications in the Fc (two amino acid substitutions: S239D and I332E) to increase the binding to Fcγ and improve the ADCC. This modification increased not only the ADCC activity but also promoted the induction of antibody-dependent cellular phagocytosis (ADCP) [76] (Figure 1B).

##### Complement-Dependent Cytotoxicity

Many therapeutic mAbs used in the conventional treatment against cancer can promote the activation of the complement classical pathway (CDC), specifically those with the IgG1 isotype. IgG1 antibodies can simultaneously promote the activation of receptors in macrophages and NK cells (Fcγ); at the same time, they regulate CDC, to which most of the therapeutic mAb try to preserve the Fc region of the IgG1. The mAbs were able to bind to the tumoral antigens expressed in the membrane of the target cell; thereafter, C1q was able to bind to the Fc region of the antibody for the activation of the proteolytic process, which then enabled the binding of other complement factors until poly-C9 was attached to the target cell for the formation of the membrane-attack-complex (MAC) [77].

As an example, Rituximab could promote synergy between ADCC (mediated by NK cells), ADCP (mediated by macrophages), and CDC [78]. Other antibodies, such as Naxitamab, also promoted this mechanism. The target molecule of Naxitamab was the glycolipid GD2, a disialoganglioside overexpressed in neuroblastoma and other neuroectodermal cells, including the central nervous system and peripheral nerves. During in vitro studies, Naxitamab was able to bind to GD2 at the cellular surface and induced CDC and ADCC [79] (Figure 1C).

##### Antibody-Dependent Cellular Phagocytosis

ADCP is the biological function mediated by the binding of Fc with the FcγRI receptor expressed in macrophages, neutrophils, and eosinophils. ADCP is the mechanism by which the antibodies opsonize the tumor cell for its internalization and degradation in the phagosome. In general, it has been observed that antibodies that induced ADCC (for example, Tafasitamab) could promote ADCP, which was associated with the production of gamma-interferon (IFN-γ) by NK cells that induced the expression of the FcγRI in polymorphonuclear cells, thus, promoting phagocytosis [80] (Figure 1D). Antibodies as Daratumumab could promote several effector mechanisms in cancer cells, such as ADCC, ADCP, CDC, apoptosis, and the modulation of CD38 enzyme activities. Daratumumab was the first fully human IgG1-κ against the C-terminal loop in the residues 189–202 and 223–236 of CD38. This antibody was approved for the treatment of multiple myeloma, and it is expressed at low levels in normal lymphoid cells, myeloid cells, and some non-hematopoietic tissues [81].

#### 3.2.2. Conjugated Antibodies

Another fascinating application of mAbs has been their use as vehicles in the transport of drugs due to their specificity and high affinity. The use of antibody-drug conjugates (ADC) arose from the need to enhance the antitumoral effects of conventional treatments, taking advantage of their specificity to target antigens in order to increase the antitumoral activity. During ADC, different effector molecules (cytotoxic agents, toxins from bacteria, proteins, plants, and radiopharmaceutical agents) promoted cellular death after binding to and internalizing antibodies [82].

**Figure 2:**
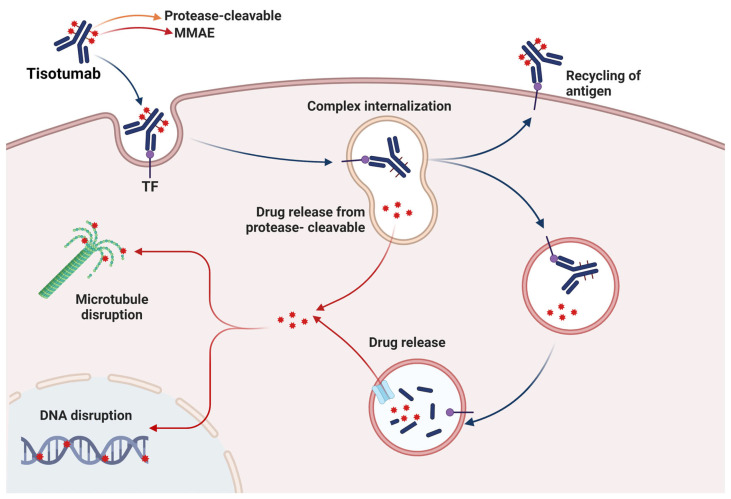
Mechanism of action of the ADC Tisotumab vedotin-tftv. Tisotumab vedotin-tftv targets and blocks TF, which later is internalized and enters the lysosome-mediated intracellular trafficking. Then, it is enzymatically degraded for the intracellular release of MMAE, which promotes cellular death by microtubule disruption. In addition, releasing MMAE into the tumoral microenvironment promotes the apoptosis of neighboring cancer cells. Red symbol represents the drug released. Image created in BioRender (www.biorender.com, accessed on 20 January 2023).

Tisotumab vedotin-tftv has been a successful ADC against the tissue factor (TF) (coagulation pathway), which performs an essential role as a receptor in signaling pathways related to cancer development. This ADC is a human IgG1 conjugated to a small molecule of monomethyl auristatin E (MMAE), a disruptor agent of microtubules. The effector mechanism of Tisotumab vedotin-tftv was the binding of the antibody to TF expressed in the tumor cells, the internalization of the ADC-TF, and the release of the MMAE by proteolytic cleavage. Later, MMAE disrupts the microtubule network in the actively dividing cells, which stops the cell cycle and induce cellular death by apoptosis. Additionally, Tisotumab vedotin-tftv can promote ADCP and ADCC by the Fc region of the antibody [83] (Figure 2).

#### 3.2.3. Disadvantages of mAb-Based Therapy

Unfortunately, only some of the mAbs have been as successful as Rituximab and other therapeutic mAbs approved by the FDA. One of the major inconveniences in therapeutic mAbs is the development of drug resistance, which increases the need to improve the knowledge of their mechanisms of action. Modifications could overcome this resistance in the conjugation with other compounds, changes in the Fc region to enhance NK cells and macrophages activation, or their use as support during conventional therapy [84,85].

On the other hand, mAbs are multimeric proteins with a molecular weight of 150 kDa, and they contain disulfide bonds and N-linked glycans as posttranslational modifications. In addition, for their in vitro production, they require sophisticated eukaryotic machinery, which increases the concentration of the antibody required during the treatment, making them inaccessible to all the patients. For this purpose, several strategies have been developed for cost reduction in commercial antibodies, such as Rituximab [86]. Historically, the first therapeutic mAbs derived from mice resulted in side effects, such as immunogenicity and poor immune response, limiting their clinical use. Currently, this disadvantage has been circumvented using biotechnological techniques that enabled the translation of the murine Fc into a fully human Fc or the complete deletion of this region for the generation of other formats of antibodies [87].

## 4. Single-Chain Variable Fragments (scFv)

Due to biotechnological advances, in 1991, it was possible to clone the Ig genes for the first time [88]. As a result, today, many formats of antibodies have been generated from these genes, which can be expressed in eucaryotic and prokaryotic expression vectors. This technology has enabled the production of recombinant versions of any antibody with reproducibility at a lower cost and shorter time, which has overcome the production problems associated with the hybridoma method [89]. The manipulation of the antibodies and the design of new fragments have broadened the possible medical applications. One of the most widely used antibody formats in medicine is scFv, which has been used as a tool in the treatment and diagnosis of cancer and research concerning novel biomarkers. The scFv has been shown to be mono and multi-specific, with greater functional affinity, better tissue retention, and effector functions [90].

### 4.1. Structure and Function

**Figure 3:**
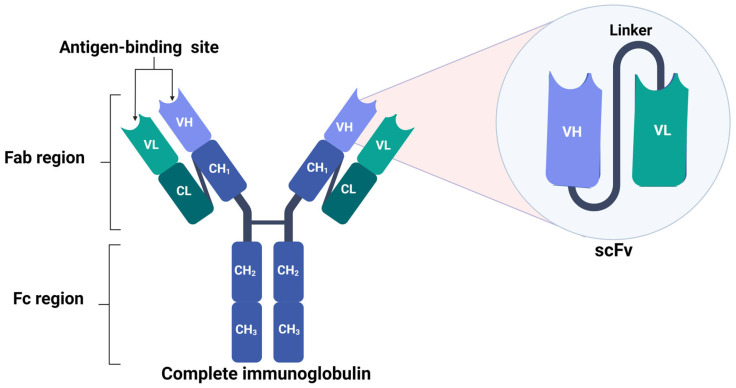
Structure of scFv. scFv structure is composed of the VH and VL chains of the complete antibody; both segments are linked with a flexible linker for the conservation of the antigen-binding site. Image created in BioRender (www.biorender.com, accessed on 20 January 2023).

As compared to complete Ig, scFvs are smaller, single polypeptides of 25 kDa that are formed by joining the V_H_ and V_L_ domains of complete Ig [91]. These antibodies are ideal for diagnostic and therapeutic applications; however, the weak binding of the variable regions makes them unstable. Therefore, to improve their stability, flexible peptide sequences (linkers) have been inserted between the V_H_ and V_L_ regions, enabling the intramolecular pairing of both domains in order to form a functional antibody-binding site [4] (Figure 3). The linker length was typically 10–25 amino acids with Glu-Lys sequences to increase solubility or Gly-Ser to increase flexibility. It was previously reported that the length and the composition of the linker are essential in the correct folding of the protein; for example, Gly-Ser linkers had short side chains that provided conformational flexibility and slight immunogenicity, while serine improved solubility [92].

As compared to complete Ig, scFv are small molecules lacking the Fc region that maintains an antigen-binding site [93,94,95]. Another difference between full-length mAbs and scFv is the presence of one glycosylation site in the C_H_2 domain of the heavy chain, which yielded stability, prevented aggregation, and promoted effector functions [96], while scFv was not glycosylated; consequently, they were easier and inexpensive to produce in microbial systems, such as *Escherichia coli* [97]. It was shown that it was possible to produce scFv from the mRNA derived from antibody-producing hybridomas; this method preserved the antigen-binding ability and increased the sensitivity, as compared to the parental hybrid cell [98,99,100].

### 4.2. Mechanisms of Action of scFv in Cancer Therapy

Although the FDA has only approved two scFv for therapeutic purposes, many are still in the research-and-development phase. To perform an action mechanism against tumor cells, scFv must be coupled with drugs, antibodies, or immune cells, due to their lack of the Fc domain. Other disadvantages associated with the effector mechanisms of ScFv have included their low thermostability and aggregation increased the risk of immunogenicity and a shorter half-life [101].

#### 4.2.1. T-Cell—Engaging CD3-Bispecific scFv Antibodies

**Figure 4:**
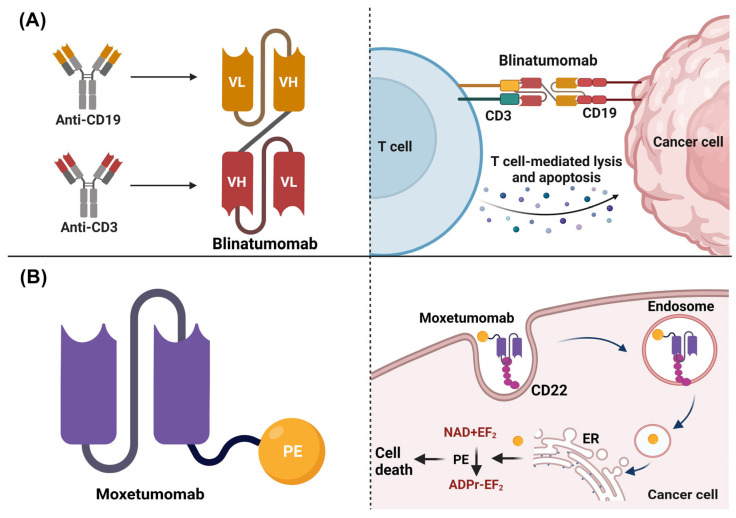
Structure and mechanisms of action of scFv approved by the FDA. (**A**) Blinatumomab structure and mechanism of action. It is composed of variable fragments of bivalent bispecific antibodies linked together. Blinatumomab stimulates a synapse between the CD3+ T cell and the CD19+ tumoral target cells, promoting the upregulation of adhesion molecules, production of cytolytic proteins, and the release of pro-inflammatory cytokines that conveyed to cellular lysis and apoptosis of the CD19+ cells. (**B**) Moxetumomab pasudotox structure and mechanism of action. It involves an anti-CD22 scFv linked to Pseudomonas exotoxin A PE38 by a peptide bond to VH. Moxetumomab binds to CD22 overexpressed in malignant B cells; later, the complex Moxetumomab-CD22 is internalized by endocytosis. Finally, PE38 catalyzes the ADP-ribosylation of the diphthamide residue in EF-2, which promotes a reduction in the levels of the antiapoptotic protein Mcl-1 and increases the apoptotic rate. Image created in BioRender (www.biorender.com, accessed on 20 January 2023).

Different strategies have been proposed to enhance the antitumoral activity and overcome the limitations associated with the use of scFv in therapy. For example, Blinatumomab was the first scFv approved by the FDA to treat relapsed or refractory Philadelphia chromosome-negative B-cell acute lymphoblastic leukemia (LLA R/R). Blinatumomab is bispecific and consists of the union of two scFv, one targeting CD19 and the other CD3 in T cells. This scFv stimulated a synapse between the CD3+ T cell and the CD19+ tumoral target cells, thus, promoting the upregulation of adhesion molecules, the production of cytolytic proteins, and the release of pro-inflammatory cytokines, which then conveyed the cellular lysis and the apoptosis of the CD19+ expressing cells (Figure 4A) [102]. Other scFvs under development have also used a bispecific structure, such as an scFv that recognized CD3+ T cells and the homolog 4 of B7 (B7-H4), a molecule associated with immune checkpoints that negatively regulate immune responses and is overexpressed in human cancers, such as breast cancer [103].

#### 4.2.2. Toxin-Conjugate scFv

Moxetumomab pasudotox was approved for treating hairy cell leukemia. This scFv was constructed using an anti-CD22 monoclonal antibody fused with a 38 kDa fragment derived from the exotoxin A PE38 of *Pseudomonas*. A single molecule of exotoxin could induce the death of tumor cells, as compared to chemotherapy drugs, in which around 105 molecules would be needed to induce the same effect [104]. The action mechanism of Moxetumomab consisted of the binding of the scFv to CD22, a surface receptor overexpressed in malignant B cells; later, the complex Moxetumomab-CD22 could be internalized by endocytosis. Finally, PE38 catalyzed the ADP-ribosylation of the diphthamide residue in the elongation factor 2 (EF-2), which promoted a reduction in the levels of the antiapoptotic protein Mcl-1 (myeloid cell leukemia 1) and increased the apoptotic rate (Figure 4B) [105].

Though there are only a few cancer treatments based on the use of scFvs approved by the FDA, numerous therapeutic strategies in different phases of development have been reported on the website https://clinicaltrials.gov/, accessed on 20 January 2023 (Table 2).

Given the success of Moxetumomab, other scFvs coupled to toxins are currently under development, including an immunotoxin linked to an scFv with high binding affinity for glioblastoma multiforme (GBM) cells that express EGFRwt and EGFRvIII [133]. In addition to toxins, other antitumoral drugs have been used, such as lidamycin, composed of enediyne chromophore with extremely potent cytotoxicity, which is currently in phase II clinical trial [140].

#### 4.2.3. Chimeric Antigen Receptor (CAR) T-Cells

**Figure 5:**
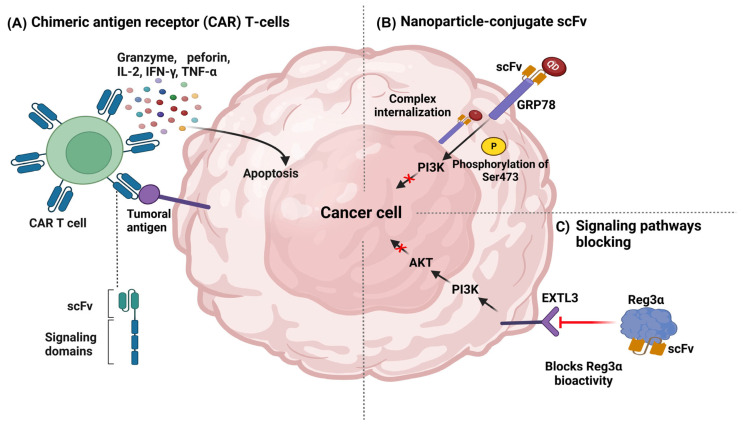
Mechanisms of action of scFv in cancer therapy. (**A**) Chimeric antigen receptor (CAR) T-cells. (**B**) Nanoparticle-conjugate scFv. (**C**) Biological activity blocking. IL-2: Interleukin 2, IFN γ: Gamma Interferon, TNF-α: Tumor Necrosis Factor Alpha.

Another promising approach of scFv during cancer treatment was their expression in T cells as chimeric antigen receptors (CAR). These novel T-cell receptors were genetically engineered to combine the extracellular antibody binding and intracellular signaling properties of CD8+ T cells, redirecting their cytotoxic capacity toward tumor cells. One of the main advantages of this strategy is that modified cells from the patient were reinfused after the genetic modification of the T-cell receptor (TCR). Afterward, chimeric receptors were able to bind to their specific ligands in the tumor cells and trigger the signal activation for activating CD8+ T cells and releasing cytokines, chemokines, and proteases [103] (Figure 5A). This type of therapy was one of the most promising, with several clinical trials in development, including for pancreatic cancer [141,142], ovarian cancer [143], and leukemia, in which CAR T cells were directed against targets such as CD70 [144,145] and CD19 [146,147] (Table 2).

#### 4.2.4. Nanoparticle-Conjugate scFv

Over time, different applications of nanoparticles in cancer, specifically for treatment, have been explored, considering advantageous characteristics, such as biocompatibility, reduced toxicity, stability, improved permeability and retention, and precise targeting. As compared with traditional delivery systems, nanoparticles possessed exceptional physical properties and a unique design that could efficiently penetrate the hypoxic tumor microenvironment and promote the effector mechanism in tumor cells [111].

Nanoparticles could be directed to the tumor site by coupling with antibodies or scFv, which could easily enter the tumor. Many nanoparticles were able to carry drugs due to their hydrophobic properties, such as liposomes. A relevant application of liposomes in colorectal cancer was via the joint administration with irinotecan, a chemotherapy drug, and an anti-FAP (fibroblast activating protein) scFv. The liposomes were modified with the ligand tripeptide motif Arg-Gly-Asp (RGD) (which binds to integrin receptors on the surface of tumor cells) and the cationic peptide 9-arginine (R9), which contributes to cell penetration and lysosomal escape due to its positive charge [148]. Some nanoparticles, such as quantum dots (QDs), emit photoluminescence and have often been used in imaging systems for tumor localization. In addition, QDs could be coupled with the scFv targeting GRP78 (a membranal protein), internalized by tumor cells, and upregulated by the phosphorylation of ser473 in AKT, thus inhibiting the tumor growth of breast cancer in a xenograft model [149] (Figure 5B).

#### 4.2.5. Blockade of Signaling Pathways and Biological Activity

One mechanism of action of therapeutic mAbs that does not require an Fc region is the blockade of cell surface receptors and molecules involved in activating signaling cascades associated with malignant progression. In this mechanism of action, the scFv was able to bind to the target molecule, and performed an antagonistic role, avoiding its interaction with ligands. For example, the scFv directed against activated the leukocyte cell adhesion molecule (ALCAM), which is involved in the development of tamoxifen resistance, an endocrine therapeutic agent that antagonizes the proliferative effect of estrogen in breast cancer tumor cells and promotes invasion, migration, and metastasis in ER+ cells. The anti-ALCAM scFv pretreatment enhanced the antiproliferative effects of tamoxifen against resistant cell lines, thus reducing migration and invasion [150].

In colorectal cancer (CRC), scFv targeted the regenerative protein 3α (Reg3α), a trophic factor that stimulates proliferation and neogenesis. The scFv was able to bind to Reg3α and suppress the cellular proliferation [151] (Figure 5C) by blocking the exostosin-like 3 (EXTL3)-PI3K-Akt signaling pathway, where EXTL3 performs a role as a receptor [152]. Additionally, scFv against immune checkpoints, such as PD-L1/PD-1 has been produced to inhibit these receptors [153].

### 4.3. In Situ Delivery of scFv Using Vectors

**Figure 6:**
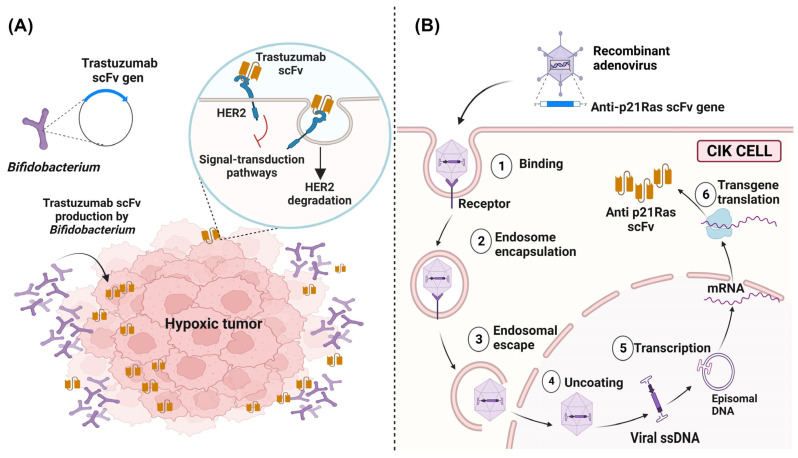
In situ delivering of scFv to tumor sites by vectors. (**A**) In situ delivery and production system of Trastuzumab scFv by Gene-Engineered *Bifidobacterium*. *Bifidobacterium*, a strict anaerobic bacterium, explicitly targets the hypoxic environment of tumors, being a transformed bacterium that expresses anti-HER2 scFv, and this antibody blocks the HER2-mediated signaling pathways. (**B**) The administration system of an anti-p21Ras scFv by transfected CIK cells. Recombinant adenoviruses loaded with the anti-p21Ras scFv gene can enter CIK cells, replicate, and intracellularly express anti-p21Ras scFv.

A novel strategy proposed in recent years was the transport of scFv directly to tumor sites by using transformed vectors, such as bacteria and viruses. In bacteria, there has been a particular interest due to their innate mobility that enables movement away from the vasculature in order to penetrate the hypoxic regions of the tumor. In addition, bacteria could proliferate and produce scFv in situ to solve the problem commonly faced with chemotherapeutics that could only reach the vascularized outer edges of the tumor but could not reach the hypoxic core [154]. For example, it was possible to construct plasmids containing the variable region sequence of the trastuzumab (anti-HER2) mAb for the transformation of *Bifidobacterium* strains (a bacterium that could be safely and selectively accumulated under hypoxic conditions) for the in situ production of Trastuzumab scFv at the tumor site and the inhibition of HER2-mediated signaling pathways [155] (Figure 6A).

Adenoviruses could transfect cells and guarantee delivery to the patient without being detected by the immune system. They also have low toxicity, and the intravenous administration of the adenovirus showed limited antitumoral activity in vivo. To enhance adenovirus delivery, cytokine-induced killer (CIK) cells have been commonly used as a secondary vector for transport to the tumor site. The use of this system for the delivery and release of scFv demonstrated an increase in the antitumoral activity within only a few days after treatment, during which the adenovirus and scFv could be detected exclusively in tumoral tissues [156]. An example of this system included the scFv anti-p21 Ras successfully used in several cancer xenograft models with mutations in the Ras gene, such as colorectal cancer [157], lung cancer [158], and liver cancer [159] (Figure 6B). Other alternative vector strategies for delivering scFvs to the tumor cells included “cell-penetrating peptides” (CPPs), which are natural or synthetic peptides with the ability to interact with cellular membranes for the internalization of cells and effective intracellular delivery. CPPs had low cytotoxicity and immunogenicity [160] and have been widely used as vehicles for the administration of scFv with a Ras-blocking effect in colon cancer [161] and lung cancer [162]; these peptides improved the intracellular administration and orientation, thus favoring the antitumoral activity of the scFv.

### 4.4. Advantages and Limitations of scFv

The use of mAbs during immunotherapy in cancer has proved to be highly beneficial in cancer immunotherapy, as compared to scFv. As a result, there have been more mAbs approved to be applied in cancer immunotherapy than scFv. This is due to, among other things, conserving the Fc region, which could induce a series of effector mechanisms that contribute to the eradication of neoplastic cells [3]. However, the conservation of the Fc region proved to be a challenge for the first monoclonal antibodies produced since its murine origin provoked an immune response against this region in patients, which then led to the production of anti-drug antibodies; therefore, murine mAbs failed to kill cancer cells [163]. A solution to the problem of an anti-murine antibody immune response was to humanize these molecules, that is, the total or partial replacement of the murine regions by human sequences [164]. A desirable feature of conventional antibodies in cancer treatment is that they have long half-lives, allowing them to adequately exert their mechanism of action. As compared to these, it has been observed that recombinant fragments with a size of less than 60 kDa, as is the case of scFv, have limitations due to their short half-lives since they are rapidly eliminated in the kidneys since their size of 25 kDa is below the glomerular filtration threshold [165]. In general, the reduced size and the lack of the Fc domain of scFv antibody constructs result in faster pharmacokinetics and potentially more homogeneous tumor penetration relative to IgG molecules. Despite these potential advantages over IgG complete, the use of scFv has been limited since the total tumor uptake is low, thus requiring the administration of a higher dose, which then results in a large accumulation occurring in these organs and can cause kidney damage [166].

Even though the size of scFv is a limitation in cancer treatment, it turns out to be an advantage for clinical diagnoses. When performing techniques, such as magnetic resonance imaging, which requires labeled antibodies that specifically bind to tumor biomarkers, it is convenient that the labeled antibody is rapidly eliminated from the body when the label is toxic or harmful. A series of potential scFvs capable of binding to different tumor antigens in a specific manner that enables the visualization of tumors and cancer cells by imaging techniques were reported. It has been shown through in vivo experiments that when comparing the effectiveness of these antibody fragments with conventional mAbs, scFvs reached the tumor site faster and remained in the body for less time than whole antibodies [131].

One of the most relevant advantages of scFv, as compared to conventional mAbs, is their low production cost; given that scFv is easily produced in bacterial systems, this type of culture is relatively easy, inexpensive, and fast, as compared to the technique based on the production and cultivation of hybridomas. Therefore, an unlimited source of antibodies can be obtained at a larger scale [167].

## 5. Conclusions

The use of monoclonal antibodies and alternative formats, such as scFv, applied to the diagnosis and treatment of cancer represents a novel alternative. These methods have overcome limitations in conventional therapies, such as damage to healthy cells, relapse, and drug resistance. However, it is necessary to explore the characteristics and properties of each molecule to understand how to apply them in specific cases. Complete monoclonal antibodies can be used to activate biological functions associated with the Fc, such as ADCC, CDC, ADCP, and signal blocking, and they can be used for CAR-T cell therapy or the development of antibody-drug conjugates. scFv are small molecules that can quickly reach and penetrate the tumor; however, this characteristic is associated with shorter half-lives since they can be easily eliminated by the kidneys; therefore, a higher concentration needs to be administrated over short periods. The use of scFv is not only limited to treatment, as it can also be used for imaging diagnostics. Due to the lack of the Fc region, scFv cannot perform the effector mechanisms of a conventional mAb. Thus, this limits their ability to block receptors and signaling pathways, for which it is necessary to combine them with drug-based therapies or T cells. Still, much research is ongoing to overcome the limitations and enhance the biological functions of both molecules. However, both are promising biotherapeutics that offer novel insights into cancer treatments.

## Figures and Tables

**Table 1:** Monoclonal antibodies approved by the FDA (www.fda.gov/, accessed on 20 January 2023).

mAb Name	Target/Epitope	Antibody Kind	Cancer Kind	Year Approval
Rituximab	CD20/169-PANPSE-174 and 183-CYSIQ-187 [22]	Chimeric IgG1	Non-Hodgkin lymphoma	1997
Trastuzumab	HER2 ^1^/extracellular domain [28]	Humanized IgG1	Breast	1998
Alemtuzumab	CD52/C-terminal with part of the GPI anchor [29]	Humanized IgG1	Chronic myeloid leukemia	2001
Ibritumomab tiuxetan	CD20/Same as Rituximab [30]	Murine IgG1	Non-Hodgkin lymphoma	2002
Cetuximab	EGFR/Domain III amino acids 334–504 [31]	Chimeric IgG1	Colorectal	2004
Bevacizumab	VEGF-A/Hairpin loop (β5–turn–β6) and β2–α2–β3 [32]	Humanized IgG1	Colorectal	2004
Panitumumab	EGFR/Domain III, P349, P362, D355, F412 and I438 [33]	Human IgG2	Colorectal	2006
Ofatumumab	CD20/FLKMESLNFIRAHT region [34]	Human IgG1	Chronic lymphocytic leukemia	2009
Ipilimumab	CTLA-4/front β-sheet [35]	Human IgG1	Metastatic melanoma	2011
Brentuximab vedotin	CD30/Extracellular domain [36]	Chimeric IgG1	Hodgkin lymphoma, systemic anaplastic large cell lymphoma	2011
Pertuzumab	HER2/Extracellular domain II [37]	Humanized IgG1	Breast	2012
Obinutuzumab	CD20/Large extracellular loop (172–176 region) [30]	Humanized IgG1 Glycoengineered	Chronic lymphocytic leukemia	2013
Ramucirumab	VEGFR2/Domain III [38]	Human IgG1	Gastric	2014
Blinatumomab	CD19, CD3/Residues 97–107, 155–166, and 216–224 [39]	Murine bispecific tandem scFv	Acute lymphoblastic leukemia	2014
Nivolumab	PD-1/BC-loop [40]	Human IgG4	Melanoma, non-small cell lung	2014
Pembrolizumab	PD-1/C, C′, and G antiparallel beta sheets and C-C′ and F-G loops [41]	Humanized IgG4	Melanoma	2014
Necitumumab	EGFR/Domain III [42]	Human IgG1	Non-small cell lung cancer	2015
Dinutuximab	GD2 [43]	Chimeric IgG1	Neuroblastoma	2015
Daratumumab	CD38/C-terminal loop (residues 189–202 and 223–236) [44]	Human IgG1	Multiple myeloma	2015
Elotuzumab	SLAMF7/IgC2 domain [45]	Humanized IgG1	Multiple myeloma	2015
Olaratumab	PDGFRα ^2^/Extracellular domain [46]	Human IgG1	Soft tissue sarcoma	2016
Atezolizumab	PD-L1 ^3^/Beta-sheet C′ and B-C loop [41]	Humanized IgG1	Bladder	2016
Inotuzumab ozogamicin	CD22/V-like domain [47]	Humanized IgG4	Acute lymphoblastic leukemia	2017
Avelumab	PD-L1/Central beta-sheets C and F [41]	Human IgG1	Merkel cell carcinoma	2017
Gemtuzumab ozogamicin	CD33/Ig-like V-set domain [48]	Humanized IgG4	Acute myeloid leukemia	2017
Durvalumab	PD-L1/Central beta-sheets C and F [41]	Human IgG1	Bladder	2017
Cemiplimab	PD-1/BC and FG loops (N58 Glycan) [49]	Human mAb	Cutaneous squamous cell carcinoma	2018
Polatuzumab vedotin-piiq	CD79β/ARSEDRYRNPKGS [50]	Humanized IgG1	Diffuse large B-cell lymphoma	2019
Enfortumab vedotin-ejfv	Nectin-4/V-domain [51]	Human IgG1	Cancers expressing Nectin-4	2019
Sacituzumab govitecan	Trop-2/Q237-Q252 [52]	Humanized IgG1	Solid tumors	2020
Isatuximab-irfc	CD38/C-terminal loop (residues 81–90) [44]	Chimeric IgG1	Multiple myeloma	2020
Tafasitamab-cxix	CD19 [53]	Fc-modified IgG1	Diffuse large B-cell lymphoma	2020
Belantamab mafodotin-blmf	BCMA ^4^ [54]	Afucosylated IgG1	Multiple myeloma	2020
Naxitamab	GD2 [55]	Recombinant humanized IgG1	Neuroblastoma	2020
Margetuximab-cmkb	HER2/Extracellular domain [28]	Chimeric Fc-engineered IgG1	Metastatic HER2-positive breast	2020
Loncastuximab tesirine-lpyl	CD19/RB4 [56]	Humanized IgG1	Large B-cell lymphoma	2021
Amivantamab-vmjw	EGFR/Residues K443, K465, I467, S468 [57] and MET	Human Ig G1-based bispecific antibody	Metastatic non-small cell lung	2021
Dostarlimab-gxly	PD-1/PD-L1/BC, C′D and FG loops [58]	IgG4 humanized	Advanced solid tumors	2021
Tisotumab vedotin-tftv	Tissue Factor [59]	IgG1	Cervical	2021
Teclistamab-cqyv	BCMA [60]	Humanized Ig G4-proline, alanine, alanine	Multiple myeloma	2022
Mirvetuximab soravtansine-gynx	FRα ^5^ [61]	IgG1, Antibody-drug conjugate	Epithelial ovarian, fallopian tube, or peritoneal	2022
Mosunetuzumab-axgb	CD20/CD3 [62]	Bispecific CD20-directed CD3 T-cell engager	Relapsed or refractory follicular lymphoma	2022

^1^ Human Epidermal Growth Factor Receptor 2; ^2^ Programmed cell death ligand; ^3^ Platelet-derived growth factor receptor alpha; ^4^ B-cell maturation antigen; ^5^ Folate receptor-alpha.

**Table 2:** Types of scFv-based cancer treatments reported at ClinicalTrials.gov, accessed on 20 January 2023.

Kind/Treatment Name	Description	Clinical Phase	Evidence
L19-IL2 is a tumor-directed immunocytokine consisting of IL2 and scFv directed against the ED-B domain of fibronectin.	Advanced solid tumors.	I/II	Register: NCT01058538 [106].
Blinatumomab.	Relapsed or refractory B-cell precursor Philadelphia chromosome-negative acute lymphoblastic leukemia (R/R ALL).	Approved	Register: BLA 125557 [102].
CAR-T cells, where the CAR consists of a scFv directed against CD19, with three intracellular signaling domains derived from CD3 zeta, CD28, and 4-1BB.	B cell lymphoma B cell leukemia.	I/II	Register: NCT02132624 [107].
CAR-T cells targeted to CD19 by a humanized scFv.	B-cell chronic lymphocytic leukemia treatment.	I/II	Register: NCT02782351 [108].
Moxetumomab pasudotox.	Hairy cell leukemia.	Approved	Register: 1020748-57-5 [105].
CAR-T cells expressing an anti-CD19 scFv bound to TCRζ and 4-1BB signaling domains.	Multiple myeloma.	I	Register: NCT02135406 [109].
CAR-T cells expressing scFv with 41BB costimulatory domain and CD3ζ signaling domain targeting mesothelin or CD19.	Pancreatic cancer.	I	Register: NCT03497819 [110].
CAR-T cells expressing an anti-CD19 scFv.	B-cell acute lymphocytic leukemia B-cell chronic lymphocytic leukemia B cell lymphoma.	I	Register: NCT03685786 [111].
CART-19 cells transduced with a lentiviral vector to express anti-CD19 scFv.	Patients With B Cell ALL, Relapsed or Refractory, With no Available Curative Treatment Options.	II	Register: NCT02030847 [112].
Combination of radiotherapy with Darleukin, which is a fusion protein L19-IL2, composed of two fractions: L19, a scFv, linked by a flexible linker to IL2.	Stage IV non-small cell lung cancer.	II	Register: NCT03705403 [113].
Autologous T cells expressing anti-CD19 scFv chimeric antigen receptors.	B cell neoplasms, B cell lymphoma, B-cell acute lymphoblastic leukemia.	I	Register: NCT03559439 [114].
CAR-T cells expressing anti-PD-L1 scFv.	Advanced lung cancer.	I	Register: NCT03330834 [115].
CAR-T cells expressing an anti-CD276 scFv.	Solid tumors.	--	Register: NCT04691713 [116].
Autologous T cells expressing an anti-BCMA scFv coupled to TCRζ and 4-1BB signaling domains.	Multiple myeloma	I	Register: NCT02546167 [117].
CAR T cells expressing anti-BCMA scFv.	Multiple myeloma	I	Register: NCT04650724 [118].
CART19 cells transduced with a lentiviral vector to express anti-CD19 scFv.	Leukemia, Acute Lymphoblastic.	II	Register: NCT02935543 [119].
TILs/CAR-TILs with PD1 knockout and Anti-PD1/CTLA4-scFv Secreting or CARs.	Solid tumors such as liver, breast, lung, colorectal, and brain.	I	Register: NCT04842812 [120].
CAR-T cells expressing scFv with affinity for malignant tumors.	Malignant tumors in children.	I	Register: NCT04691349 [121].
Autologous T cells expressing scFv with specificity against GFRα4.	Recurrent or metastatic medullary thyroid cancer.	I	Register: NCT04877613 [122].
CART-meso cells expressing an anti-mesothelin scFv fused with TCRζ and 4-1BB costimulatory domains.	Pancreatic cancer.	--	Register: NCT03638193 [123].
CAR T cells (huMNC2-CAR44) that bind via a scFv to the extracellular domain of the cleaved form of MUC1 (called MUC1*).	Metastatic breast cancer.	I	Register: NCT04020575 [124].
CAR-T cells secreting scFv against OX40.	Lung cancer, hepatocellular carcinoma and solid tumor.	I	Register: NCT04952272 [125].
CAR-T cells that express scFv’s against PD1/CTLA4/Tigit.	Lung cancer.	I	Register: NCT03198052 [126].
CAR-T cells with Ibalizumab-derived anti-CD4 scFv and the intracellular domains of CD28 and 4-1BB coactivators fused with the CD3ζ T cell activation signaling domain.	T cell lymphoma. T cell leukemia.	I	Register: NCT03829540 [127].
CAR-T cells expressing an anti-BCMA scFv.	Multiple myeloma.	I/II	Register: NCT05066646 [128].
Autologous T cells expressing an anti-PSMA scFv, CD2 costimulatory domain, and dual-shielded with a dominant TGFβ receptor-negative domain and PD1.CD28 switch.	Metastatic prostate cancer.	I/II	Register: NCT05489991 [129].
AR-NK cells that express and secrete IL7/CCL19 and/or scFv against PD1/CTLA4/Lag3, targeting Claudin 6.	Stage IV Ovarian Cancer Treatment. Refractory testicular cancer. Recurrent endometrial cancer.	I/II	Register: NCT05410717 [130].
Autologous T cells containing anti CD19 and anti CD20 scFv coupled to CD3ζ and co-stimulatory domain 4-1BB (4-1BB).	Non-Hodgkin’s lymphoma. B cell lymphoma. Chronic lymphocytic leukemia. Small lymphocyte lymphoma.	I	Register: NCT03019055 [131].
Tetravalent IgG(H)-scFv fusion-type of bi-specific antibody (BsAb).	Solid tumor. Advanced cancer metastatic cancer. Gastric cancer. Gastroesophageal junction carcinoma adenocarcinoma of the esophagus. Pancreatic ductal adenocarcinoma.	I	Register: NCT04900818 [132].
D2C7-IT is an immunotoxin composed of a scFv with high affinity for EGFRwt and EGFRvIII.	Malignant glioma recurrent brain tumor	I	Register: NCT02303678 [133].
L19TNF is a fully human fusion protein consisting of human TNF-α fused to the L19 antibody in scFv format, specific for the extra B domain of fibronectin.	Glioblastoma.	I/II	Register: NCT04573192 [134].
CAR-T cells expressing an anti-GPRC5D scFv.	Relapsed/Refractory Multiple Myeloma Plasma cell leukemia.	I	Register: NCT05219721 [135].
BRITE is a bispecific hooker that has one scFv binding for the CD3 epsilon subunit while another scFv is directed against the hEGFRvIII epitope that is differentially expressed on the surface of tumor cells.	Malignant glioma Glioblastoma.	I	Register: NCT04903795 [136].
CAR-T cells expressing an scFv that recognizes CD19 and dual co-stimulating intracellular signaling domains (4-1BB and CD3ζ).	Recurrent Non-Hodgkin’s lymphoma. relapsed adult ALL. Recurrent Pediatric ALL.	I/II	Register: NCT03938987 [137].
Autologous Lymphoma Ig-derived scFv-chemokine DNA Vaccine.	Lymphoplasmacytic Lymphoma.	I	Register: NCT01209871 [138].
T cells expressing anti-CD123 scFv chimeric antigen receptors linked to TCRζ and 4-1BB signaling domains.	Acute myeloid leukemia, relapsed. Acute myeloid leukemia, pediatric. Refractory acute myeloid leukemia.	I	Register: NCT04678336 [139].

4-1BB: Cluster of differentiation 137; TILs: Tumor infiltration lymphocytes; IL2: Interleukin 2; PSMA: Tumor antigen prostate specific membrane antigen.

## Data Availability

Data sharing is not applicable.
